# Longitudinal studies in forensic child and adolescent psychiatry and mental health: CAPMH thematic series 2018/2019

**DOI:** 10.1186/s13034-019-0280-5

**Published:** 2019-04-11

**Authors:** Klaus Schmeck, Jörg M. Fegert, Cyril Boonmann

**Affiliations:** 1 Kinder- und Jugendpsychiatrische Forschungsabteilung, Universitäre Psychiatrische Kliniken Basel, Universität Basel, Schanzenstr. 13, 4056 Basel, Switzerland; 2 Klinik für Kinder- und Jugendpsychiatrie/Psychotherapie, Universitätsklinikum Ulm, Steinhövelstraße 5, 89075 Ulm, Germany

The 2018/2019 Child and Adolescent Psychiatry and Mental Health (CAPMH) thematic series on forensic child and adolescent psychiatry and mental health is focused on longitudinal research. Although the importance of such studies is widely recognised, they are still rare in adolescent forensic psychiatry and psychology.

Longitudinal studies like Emmy Werner’s Kauai Study [1], Michael Rutter’s Isle-of-White-Study [2], David Farrington’s Cambridge Study [3], the Great Smoky Mountains Study [4] or, above all, the Dunedin Study [5], have had and still have a substantial influence on our understanding of child psychiatric disorders and their development over time. Though mental disorders and psychopathology in general are the core of these studies, all of them have also yielded significant contributions to the understanding of the aetiology and course of delinquent behaviour. One prominent example is the distinction between life-course persistent versus adolescence-limited antisocial behaviour, a developmental taxonomy that Moffitt has proposed on the basis of findings from the Dunedin Study [6]. This important distinction has been confirmed in many studies [7], and has stimulated further research on antisocial trajectories [8].

When forensic experts write reports for the court, they try to disentangle the complex context that may have led to a person’s crime. This search for an understanding of the individual causes of a criminal act is done retrospectively, which might inform us about potential causes of the offending behaviour. However, what seems plausible in a retrospective view is often not sufficient to predict future behaviour. We, therefore, need prospective, longitudinally designed studies that broaden our knowledge about the causes of criminal behaviour, its course and its prognosis. Thus, preventing the onset of delinquency, or, if criminal behaviour already occurred, to prevent recidivism, should be in the focus of our scientific work in adolescent forensic psychiatry.

The 2018 thematic series comprises four papers:

In the first paper, Souverein et al. [9] report the results of a panel discussion at the 2018 European Association for Forensic Child and Adolescent Psychiatry, Psychology and other involved Professions (EFCAP) congress in Venice, regarding the current situation of services for delinquent youths in various European countries as well as future directions for the improvement of these services. The panel integrated the view from five European countries (Finland, Great-Britain, Italy, Netherlands and Switzerland) and formulated cross-national mission statements for adolescent forensic health care.

In the second paper Ed Hilterman et al. [10] present a longitudinal study on 5205 male juvenile offenders from the Catalan juvenile justice system. These youths received multiple SAVRY risk/need assessments over time. With the use of growth mixture modeling and multinomial logistic regression analyses four heterogeneous trajectories of offending were identified.

The next paper of Van der Pol et al. [11] describes the long-term outcome of a randomised controlled trial. In total, 109 adolescents with cannabis use disorder and comorbid problem behavior were treated with Multidimensional Family Therapy (MDFT) or Cognitive Behavior Therapy (CBT). Both treatments were found to be effective in reducing delinquent behavior. No differences were found between MDFT and CBT in the efficacy to reduce the frequency or severity of offending over a 6 years’ time period.

Finally, Collins and Grisso [12] describe the use of two short screening instruments (Massachusetts Youth Screening Instrument—Second Version [MAYSI-2] and Strengths and Difficulties Questionnaires [SDQ]) in a sample of 1259 detained boys and its ability to predict violent offending. Their results showed that the relation between MAYSI-2 and SDQ scale scores and future violent offending varied between different ethnic groups. They even found opposite relations for boys from different ethnic backgrounds. The disillusioned conclusion of the authors is that screening for psychiatric problems in boys cannot be recommended to identify those adolescents who are at risk for committing future violent crimes and that ethnic differences in the relation between psychiatric problems and future criminality have to be taken into account.

Again, like last year, the majority of authors of the 2018 thematic series is from the Netherlands. While we warmly welcome the engagement of our young Dutch colleagues we would like to stimulate researchers from other European and non-European countries to engage in research on adolescent forensic issues and to submit papers for the next edition of this thematic series.
